# The development of proxy indicators to measure workforce well-being in emergency obstetric and neonatal care settings

**DOI:** 10.7189/jogh.15.04223

**Published:** 2025-08-10

**Authors:** R Rima Jolivet, Erica Munson, Isabel Gouse, Elizabeth Chodzaza, M Elahi Chowdhury, Thierno Dieng, Samantha Lobis, Isabelle Moreira, Kate Ramsey, Caitlin Warthin, Lynn P Freedman

**Affiliations:** 1 Independent consultant, Boston, USA; 2 Averting Maternal Death and Disability Program, Columbia University Mailman School of Public Health, New York, USA; 3 Department of Midwifery, Neonatal and Reproductive Health Sciences, Kamuzu University of Health Sciences School of Maternal, Blantyre, Malawi; 4 Health Systems and Population Studies Division, International Centre for Diarrhoeal Disease Research, Dhaka, Bangladesh; 5 de Recherche et de Plaidoyer en Santé de la Reproduction, Centre Régional de Formation, Dakar, Senegal; 6 Scope Impact Oy, Helsinki, Finland

## Abstract

**Background:**

A shortage of health workers with skills to deliver high-quality routine and emergency maternal and newborn health (MNH) care hinders achievement of the Sustainable Development Goals. Health worker well-being is a determinant of the quality and experiences of care. The emergency obstetric and newborn care (EmONC) framework supports country-level planners and managers to evaluate EmONC systems. We describe a measure development activity to identify a brief set of tracer measures to monitor facility-level health worker well-being in EmONC facilities.

**Methods:**

We did an iterative, systematic, rapid scoping review of the literature on health worker well-being constructs and existing measures, complemented by focus group discussions and expert technical consultations. We systematically mapped, evaluated, and prioritised candidate items for indicator development, drawing on the literature and inputs from content experts (MNH health workers and stakeholders) and measurement experts, to select succinct, relevant, actionable tracer measures for monitoring health worker well-being at the facility, sub-national, and national levels. We chose the final items for indicator development based on clarity, sensitivity to change, interpretability, usefulness, generalisability, and reliability.

**Results:**

Burnout, lack of psychological safety, and moral distress were prioritised to serve as proxies for the whole domain of EmONC health worker well-being. We compiled three brief measures fully reflecting the operational definition for each construct, including complete metadata. Next steps include collecting pilot data and performing psychometric testing to validate the measures. Subsequently, we hope they can be implemented as tracer indicators to monitor overall facility-based health worker well-being.

**Conclusions:**

We propose these candidate indicators as proxies to raise a ‘red flag’ when health worker well-being is low. We hope that they can alert health system administrators to the need to identify and address the root causes of threats to well-being, ensuring facility readiness to deliver EmONC.

Approximately 800 women die daily during pregnancy and childbirth, along with 6700 newborns and 5082 stillbirths [1–3]. The Sustainable Development Goals (SDGs) set ambitious targets for maternal and neonatal mortality reduction (SDG 3.1 and SDG 3.2.2) [4] and call on countries to achieve universal health coverage (SDG 3.8), including universal access to quality essential health care services, by 2030 [5]. However, a critical shortage of frontline health workers with skills to deliver routine and emergency maternity care interventions hampers achievement of these SDGs [6–8]. Without a robust workforce that is adequate in both numbers and competencies, working within a strong enabling environment, universal access to high-quality care will not be achievable [9,10]. However, numerous reports document challenging and stressful working environments and conditions for maternal and newborn health (MNH) care providers in diverse global settings [11–14].

In 2009, the World Health Organization (WHO), along with the United Nations Population Fund, the United Nations Children’s Fund, and the Averting Maternal Death and Disability Program of the Columbia University Mailman School of Public Health, issued a handbook entitled ‘Monitoring emergency obstetric care – a handbook’ [15]. Focussed on high-quality emergency obstetric care as a key strategy for reducing preventable deaths, the handbook guided its assessment delivered in facilities to women experiencing complications during pregnancy and childbirth and addressed intrapartum foetal and newborn complications, although to a lesser extent. It provided guidance and indicators for evaluating the availability, use, and quality of emergency obstetric care services, particularly through monitoring tracer services known as emergency ‘signal functions’ [15]. The handbook is currently undergoing a comprehensive overhaul aimed at ‘re-visioning’ optimal guidance for health systems delivering emergency obstetric and newborn care (EmONC).

The new guidance will add more signal functions for care of small and sick newborns and an explicit focus on monitoring facility readiness as reflected in drugs, supplies and equipment in stock, adequate numbers of skilled personnel, and emergency referral. Drawing on formative research using the principles of human-centred design, the next EmONC handbook will include guidance to monitor EmONC service user experiences of care and health workforce well-being in low- and middle-income countries (LMICs) [16–19].

Interest in measuring the construct of well-being has increased over recent decades in the fields of economics [20,21], public policy, and the behavioural sciences, including psychology, sociology, and medicine [22,23]. There are various approaches to defining well-being, with consequences for its measurement. According to the WHO, well-being is a positive experiential state that applies to both individuals and societies, and is influenced by upstream social, economic, and contextual determinants [24]. Conceptually, well-being can be framed as hedonism – the experience of pleasure, eudaimonism – the fulfilment of one’s potential, or as quality of life –the presence or absence of physical, social, and psychological conditions thought to be associated with a good life [22]. Well-being can be framed subjectively, as in satisfaction of one’s desire, or objectively, as in acquisition of goods or conditions that confer advantage regardless of how one feels about them [25]. It can be described by positive constructs that comprise it and by negative constructs that threaten it [6-8]. Thus, the concept of well-being is multidimensional and complex [9,10]. Although there is no single accepted definition of worker well-being, a multidisciplinary review defined it as ‘the experience of positive perceptions and the presence of constructive conditions at work and beyond that enables workers to thrive and achieve their full potential’ [26].

Health workforce well-being has been studied in recent years, especially in high-income settings [27,28] and since the COVID-19 global pandemic [29–31]. A 2017 evidence synthesis published by the National Academy of Medicine in the USA documented direct effects associated with reduced well-being among health care workers (*e.g.* high rates of burnout, anxiety, depression, increased substance abuse disorder, and suicide) and its indirect effects on patient outcomes (*e.g.* medication errors, malpractice, and poor communication with patients) [32]. The prevalence of provider burnout is also substantial in LMICs, regions that often experience staffing and resource shortages [33,34]. Other provider experiences, such as moral distress and psychological safety, remain understudied in these settings. As findings from high-income countries may be difficult to generalise due to cultural differences and a greater imbalance between job demands and available resources [33], additional research is needed to better understand provider experiences and the associated impacts on patient outcomes in LMICs.

Reports of mistreatment of women and their offspring in health care facilities around the time of birth have been documented in recent decades [35–38], and the root causes of these occurrences in LMICs have been studied [36,39–42]. Evaluation of the systemic drivers of poor quality and poor experiences of care in LMICs draws attention to health system deficiencies and organisational factors in the maternity care workplace that negatively affect worker well-being and may be implicated in the causal chain of mistreatment [14,41,43–46]. Conversely, effective team dynamics and interprofessional collaboration are associated with improved outcomes of EmONC in LMICs [47,48].

In elevating person-centred maternity care for women and their families, the notions of person-centred health care delivery systems and system accountability take on salience [49]. The International Labor Organization declares that workers have a fundamental right to a safe and healthy working environment [50]. Since health worker well-being is a determinant of the quality of care provided and how that care is experienced by its beneficiaries [51], the ability to measure health worker well-being in maternity care facilities in LMICs has the potential to improve necessary process and outcome measures on the causal chain toward effective, high-quality, person-centred care. Thus, having indicators to measure and track maternity care workers' well-being in LMICs could promote system awareness, improvement, and accountability.

How to validly measure self-reported outcomes, such as well-being and experiences of care on a large scale, is an important area of interest. Important work in MNH measurement has explored the relationships between meaning, meaningfulness, measurability, and measurement, and how these contribute to measure validity [52]. Construct validity is the degree to which an indicator accurately reflects the concept or phenomenon it is meant to track. However, the conceptual clarity of many MNH policy-level indicators is low [53]. Authors of a review of population health indicators emphasised that ‘a concept-driven selection process should result in more methodologically sound indicators’ [54]. Likewise, a conceptual framework for validity in MNH measurement gave utmost importance to clarity of the construct for measurement, including its meaningfulness (*i.e.* relevance and significance) to stakeholders. The authors called attention to the lack of published literature addressing this aspect of indicator validity [55].

To contribute to the ‘re-visioning’ EmONC framework’s indicator set, we aim to systematically draw upon the best available evidence and expert professional opinion to identify a brief set of no more than three facility-based tracer measures that can be aggregated as facility and district level indicators, and ideally, integrated into routine health information systems to monitor EmONC health workforce well-being, including measures of both individual health worker well-being and team well-being.

## METHODS

We applied a systematic approach to identify and prioritise relevant constructs for measurement, and to articulate tracer indicators synthesised from existing validated measures that can be used to monitor them. We used a variety of data sources and methodologies to accomplish this work.

From 6 March to 24 September 2023, we conducted a structured, iterative, rapid scoping review of grey and published literature on health worker well-being constructs and existing measures. We conducted focus group discussions and expert technical consultations to assess and prioritise opinions based on their importance and relevance in LMIC settings, and to assess construct and content validity. We compiled validated items into brief measures reflecting conceptual content validity and articulated standard metadata for their transformation into facility- and district-level indicators. We developed recommendations for pilot testing and statistical validation needed before their inclusion in the updated EmONC framework, which was not possible within the scope of this activity due to a lack of funding.

**Figure 1:**
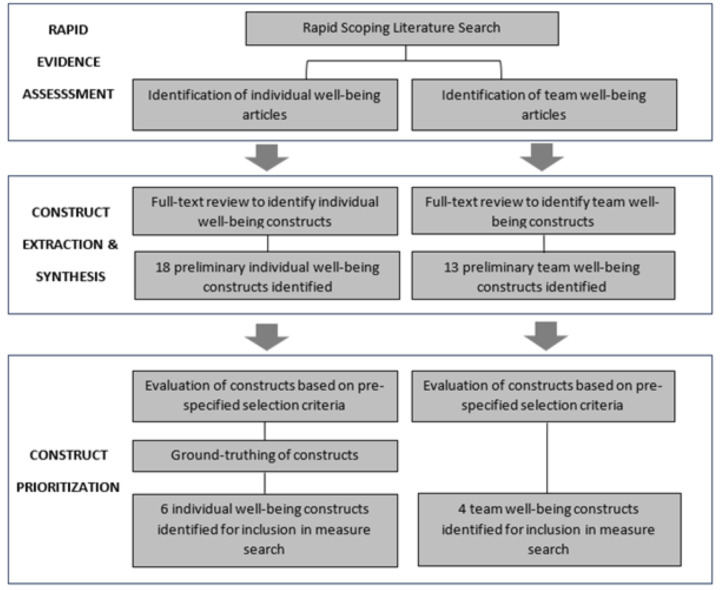
Flowchart of construct identification, critical interpretive synthesis, and prioritisation.

We created a five-phased research and development plan to guide and document progress toward our key objectives (**Box 1**). We first identified and compiled the constructs for measurement (objectives 1 and 2) (**Figure 1**), and then searched, synthesised, and assessed measures, articulated as facility-level indicators, to monitor them (objectives 3, 4, and 5).

Box 1 **Measure development approach**

**Defining constructs**

Objective 1. Identifying constructs

1.1. Implementing a systematic approach to review the grey and published literature

1.2. Compile evidence-based constructs related to individual and team well-being

1.3. Evaluate the constructs against predetermined criteria

1.4. Compile candidate constructs for indicator search or development

Objective 2. Ground-truth the constructs for measurement

2.1. Query MNH stakeholders to prioritise constructs by importance and relevance

2.2. Compile outputs to finalise the pool priority constructs for measurement

**Defining measures**

Objective 3. Identify, evaluate and synthesise measures for priority constructs

3.1. Search for existing measures for identified priority constructs

3.2. Screen identified measures against predetermined criteria

3.3. Synthesise validated items into proposed measures based on relevance and quality

3.4. Query measurement experts on construct validity of candidate measures

Objective 4. Enumerate metadata

4.1. Enumerate standard metadata for three proposed tracer measures of health worker well-being

4.2. Compile into facility-level indicators that can be aggregated to district level

Objective 5. Draft documentation

5.1. Develop recommendations for piloting and validation of the proposed measures

### Study definitions and predetermined evaluation criteria

#### Conceptualisation of constructs

Given that our primary aim was to raise the salience of the human experience of people who work in EmONC facilities both as individuals and as members of a team, we conceptualised eligible constructs as those that health workers prioritise as relevant and important to them and those that centre subjective, affective concepts of well-being, to extend human-centred care principles to the health workforce.

#### Predetermined evaluation criteria

We developed criteria *a priori* for evaluating both constructs and their measures, based on previous measure development efforts [54,56–59] (**Box 2**).

Box 2 **Construct and measure evaluation criteria**

**Construct evaluation criteria**

1. Relevance: the construct is directly described by health workers as important to them and linked to their well-being at work, and is something that applies broadly across diverse low- and middle-income countries and contexts.

2. Importance: the construct is something that matters and is seen as valuable to the people who can make actionable decisions in response to health workers’ needs (*i.e.* facility and district health managers, policymakers, government officials, and so on). In addition, doing something about this is something that health workers and decision makers feel would ‘make a difference’ for health workers well-being.

**Measure evaluation criteria**

1. Clarity of focus and meaning: the measure is unambiguous and reflects or represents the object of the evaluation/component accurately.

2. Sensitivity to change: the measure can be used longitudinally to assess the impact of interventions or changes over time.

3. Interpretability: the measure score or value is easy to interpret, has existing norms or benchmarks.

4. Comparability/consistency/ reliability: the measure is applicable in diverse settings and is relevant to diverse groups of health workers (i.e. can be applied to both physicians and nurses within a health system).

5. Usefulness: the measure points to a response by organisational leadership.

### Identifying constructs for measurement of individual and team health worker well-being

#### Rapid scoping literature search for articles related to individual and team well-being

We conducted a rapid scoping review of the evidence and critical interpretive synthesis to identify and categorise essential constructs related to health worker well-being in the grey and peer-reviewed literature, using PubMed, Google Scholar, and Google as databases. We chose the rapid evidence assessment methodology because it uses the same approach and principles as a systematic review and allowed us to undertake a structured assessment of the topic with an abridged breadth to accommodate pragmatic considerations while still reaching saturation [60–62]. Furthermore, we used the PRISMA checklist to guide and organise the reporting of our rapid review process and results [63]. We conducted two separate reviews: the first focussed on constructs of individual well-being and the second on constructs reflecting team well-being in the context of health care.

We conducted an iterative search in PubMed using MeSH terms related to individual health worker well-being, framed either positively (reflecting well-being) or negatively (reflecting a barrier or lack of well-being). We iteratively added related search terms that emerged in a snowball search strategy. Three rounds of searches were conducted, with each search building on MeSH terms identified through the previous search, with saturation reached after the conclusion of the third search. Following the systematic search in PubMed, the same search terms for individual well-being that emerged from PubMed were entered into Google Scholar and Google (Box S1 in the **Online Supplementary Document**). Finally, we conducted hand searches of the reference lists of articles thus identified.

To identify constructs related to team well-being, a second iterative search was conducted in PubMed using the same procedure, starting with ‘health’, ‘team’, ‘EmONC’, ‘well-being’, and their variations, and then adding terms as they emerged to amplify the constructs comprised within the basic concept of health worker team well-being as evidenced in the literature (Box S2 in the **Online Supplementary Document**).

**Figure 2:**
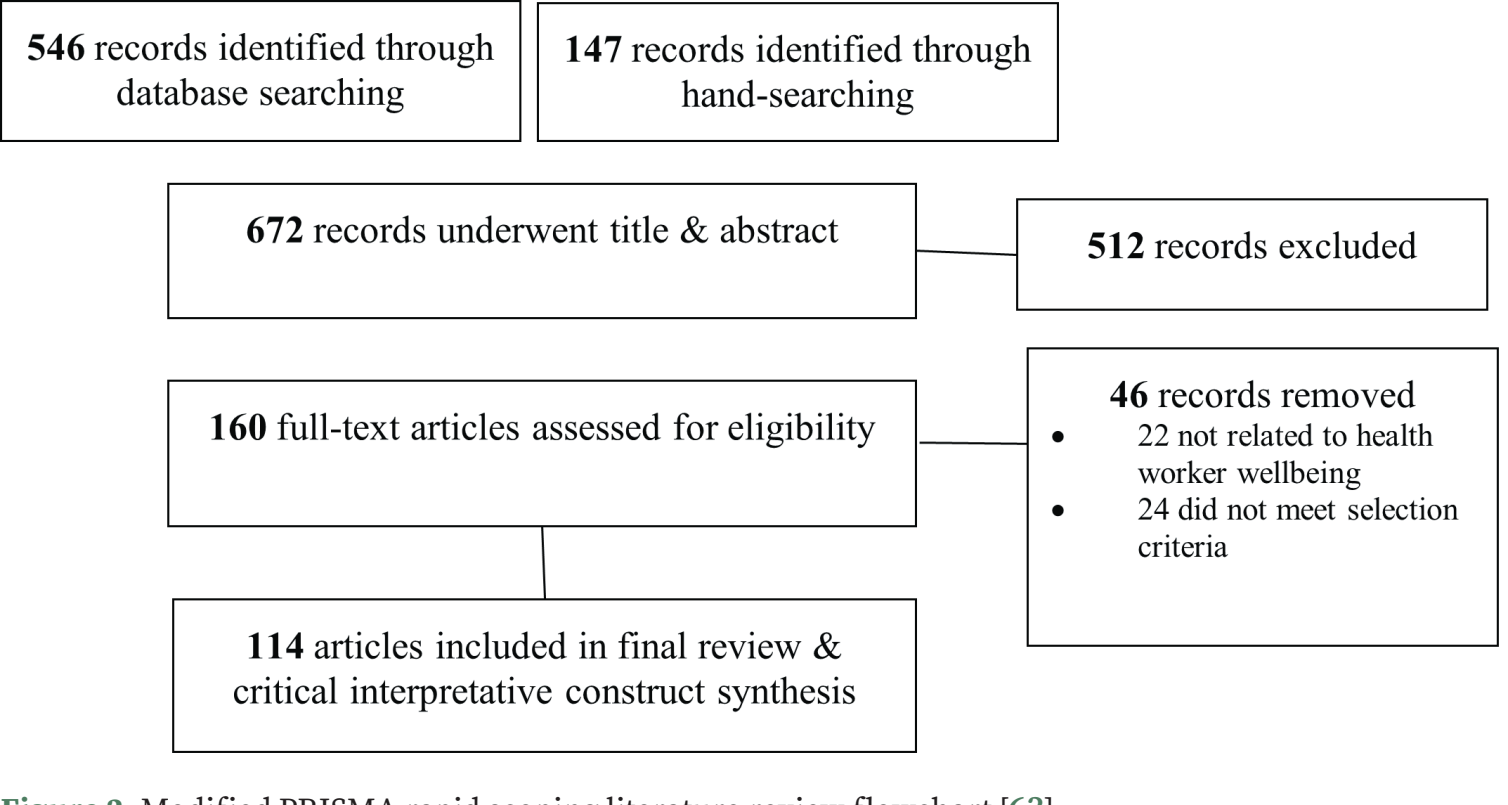
Modified PRISMA rapid scoping literature review flowchart [63].

We filtered both searches for meta-analyses and review articles published in English in the past ten years, which resulted in 114 included articles (**Figure 2**).

#### Synthesis of individual and team well-being candidate constructs

Following the rapid scoping literature review, we used critical interpretive synthesis, combining induction and interpretation to organise constructs and subconstructs identified in the primary studies into the higher-order theoretical structure of health worker well-being [64–66]. We reviewed all articles, extracted, analysed (using thematic exploration), and categorised constructs related to individual and team well-being. We compiled 18 constructs related to individual and 13 constructs related to team well-being (**Table 1**).

**Table 1:** Identified set of individual and team well-being constructs from the literature

Individual well-being constructs	Team well-being constructs
Negative constructs	Positive constructs only
*Burnout*	*Psychological safety*
*Negative tempo*	*Shared decision-making*
*Work stress*	*Cooperating*
*Moral distress*	*Mutual trust and respect*
*Turnover intention*	*Mutual support*
*Job strain*	*Shared mission*
*Workplace conflict*	*Shared material models*
*Stagnation*	*Team climate/orientation*
*Material stress*	*Collaboration*
Positive constructs	*Positivity*
*Professional fulfilment*	*Team efficacy*
*Optimal tempo*	*Role clarity*
*Confidence*	*Effective communication*
*Meaning*	
*Personnel loyalty*	
*Job ease*	
*Psychological safety*	
*Growth opportunities*	
*Material stability*	

#### Individual well-being construct prioritisation

To systematically prioritise candidate constructs for measurement of individual well-being, we first critically evaluated and scored each construct based on our pre-specified criteria of relevance and importance. We considered the preferred framing (negative *vs.* positive) for each of the top-scoring constructs. We analysed the scores in R Studio version 4.5.0. (Posit Software, PBC, Boston, Massachusetts, USA) to identify the top constructs. We carried forward a resulting short list of eight individual well-being constructs to the ground-truthing process, including work stress, job strain, workplace conflict, confidence, growth opportunities, material stability, burnout, and personnel loyalty.

We defined ‘ground-truthing’ as getting feedback and checking assumptions from the perspectives of end users and other key stakeholders. We sought end-user perspectives through the review and secondary analysis of ‘re-visioning’ EmONC qualitative data collected from frontline EmONC health workers and managers by co-authors EC, MEC, TD, and IM in Bangladesh, Malawi, and Senegal [16–18] and from a focus group discussion at the first biennial International Maternal Newborn Health Conference (IMNHC), held in Cape Town, South Africa, from 8 to 11 May 2023.

At the IMNHC, during a ‘re-visioning’ EmONC thematic workshop led by the Averting Maternal Death and Disability Program, a session was devoted to reviewing and discussing the eight prioritised individual workforce well-being constructs. The focus group session was recorded and later transcribed. Several note-takers on-site helped capture the information shared by participants. A total of 14 people participated in the focus group session, representing sexual and reproductive health non-profit organisations, ministries of health, national government agencies, and the United Nations agencies. Following a contextual overview, we asked the participants to individually review and rank the top five constructs among the eight provided based on the same predetermined criteria for relevance and importance to health workers. The focus group facilitators collected the individual votes, and the research team tabulated them. Focus group members also voted using sticker dots on a whiteboard to launch a group discussion about the importance and relevance of the candidate constructs. We collected and considered these qualitative inputs in the final analysis.

Discussants highlighted that moral distress, a construct that had been eliminated in the first round of prioritisation, is both frequent and severe among health workers and should be considered a priority. Based on this feedback, moral distress was returned to the final list of constructs for which measures would be sought. At the end of this process, we operationalised constructs reflecting stakeholder feedback on the most critical aspects of individual health worker well-being using associated subconstructs synthesised from the literature (**Table 2**).

**Table 2:** Final list of individual well-being constructs and subconstructs for candidate measure search

Construct	Subconstruct
Burnout [67-71]	Emotional exhaustion, physical exhaustion, depersonalisation and compassion fatigue
Confidence [72,73]	Competence matches one’s role, confidence in skills needed to do one’s job, self-efficacy, belief in self/ability
Job strain [74,75]	High demand/low control over work, lack of input or control over work processes and outputs, excessive workloads, inadequate staffing, time pressure
Moral distress [76,77]	Feeling powerless at work, inability to provide quality, ethical care at work due to factors beyond one’s control (*e.g.* time pressure, lack of skills, lack of resources/staff shortages, and/or system culture/policies), related distress
Work stress [78]	Task anxiety, anxiety surrounding lack of competency, feeling ill-prepared, unsupported, and unable to perform job role/tasks, related frustration
Workplace conflict [79,80]	Poor conflict management at work, incivility, bullying, verbal abuse, lack of support (from superiors or peers), physical security fears or violence including reprisal from patients

#### Team well-being construct prioritisation

Based on our *a priori* conceptualisation of health worker well-being, we prioritised constructs centring the subjective and affective dimensions of well-being among members of a health care team over constructs that centred performative aspects of teamwork.

We evaluated constructs against predetermined criteria for relevance and importance. Thus, from the 13 constructs identified, four constructs were prioritised to represent team well-being: role clarity, psychological safety, effective communication, and team efficacy. We operationalised the final list of prioritised team constructs using critical interpretive synthesis of associated subconstructs synthesised from the literature (**Table 3**).

**Table 3:** Final list of team well-being constructs and subconstructs for candidate measure search

Construct	Subconstruct
Effective communication [81-83]	Effective information exchange/knowledge sharing, effective feedback mechanisms (debriefing, reflexivity, closed loop communication), effective decision making
Psychological safety [84-88]	Feeling safe to speak up, feeling safe to learn, including from mistakes, feeling safe to take interpersonal risks
Role clarity [89,90]	Mutual understanding of roles and responsibilities (clarity about others’ roles), clear job descriptions and expectations (clarity about one’s role), clear understanding of one’s role within the team (team clarity)
Team efficacy [59]	Belief in the team’s ability to achieve goals, belief in the team’s ability to function well as a team

Due to time and resource constraints, constructs related to team well-being did not undergo an external ground truthing process at this stage.

### Identifying measures of individual and team health worker well-being

#### Search for measures of individual and team well-being

**Figure 3:**
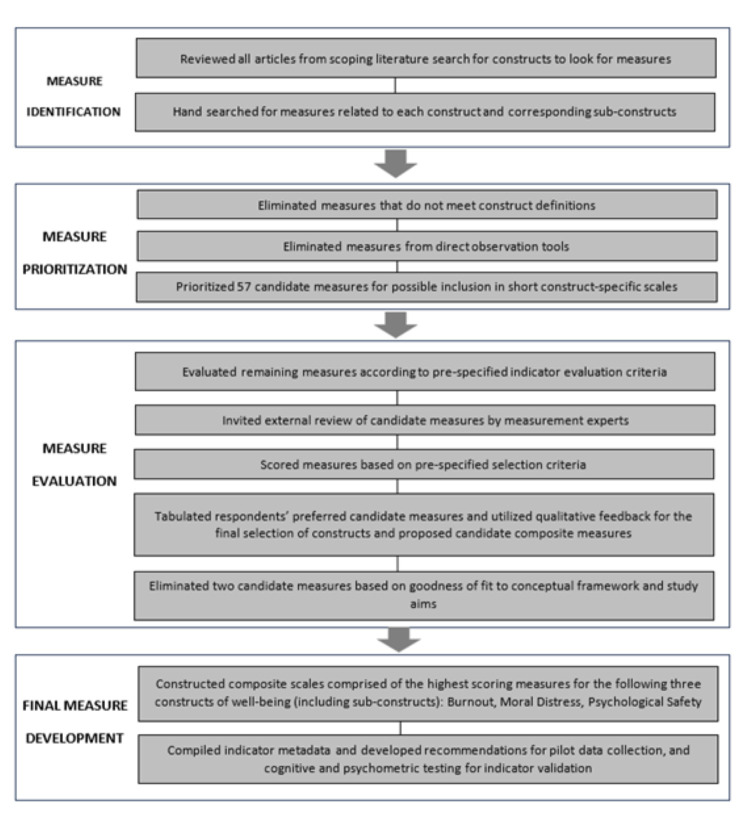
Flowchart of measure identification, prioritisation, evaluation, and final selection.

Having completed a rigorous, structured process to identify and ground-truth constructs of health worker well-being that are relevant and important in LMICs, we set out to identify existing measures of these constructs (**Figure 3**).

We identified the potential measures for indicator development using a purposive three-step search process. First, we reviewed relevant compendia of measures from leading measurement organisations such as the WHO and MEASURE Evaluation. Second, we re-reviewed articles identified in the scoping review of relevant constructs to search for measurement tools. Third, we conducted a hand search for each construct and the corresponding subconstructs to identify measurement tools that had not been previously captured.

Although we aimed to identify facility indicators, our search found only five aggregate facility-level indicators matching our constructs, and none specified which instrument should be used to collect the data needed to compile them. For example, ‘The proportion of staff who have been oriented to their functions, roles and responsibilities in the facility or unit to which they are assigned’ is a facility-level indicator that aggregates individual data on role clarity among health workers, but it does not specify how the component data from health workers should be measured [91,92]. Therefore, we were obliged to evaluate individual-level, self-reported measures captured in validated scales (*i.e.* single items, multiple-item subscales, and complete surveys), for later compilation and aggregation into facility-level indicators.

#### Prioritisation of measures

Following measure identification, RRJ, IG, and EM screened and eliminated all those that did not reflect construct definitions. We further eliminated direct observation tools that would be infeasible to convert into facility-level indicators for routine monitoring. Two reviewers independently reviewed all instruments and resolved differences by discussion. We prioritised, in order, validated single-item measures, validated subscales, and items from validated instruments.

After completing initial screening, RRJ, IG, and EM systematically evaluated 109 candidate measures for quality according to the pre-specified measure evaluation criteria (**Box 2**) and ranked the top three candidate measures for each construct. A total score for each measure was calculated based on a summative score of the evaluation criteria plus a weighted score of the ranking results. Following data analysis, we retained and submitted the two measures with the highest total scores for each subconstruct for external review. Thus, 57 candidate measures were prioritised for possible compilation into brief scales that would demonstrate content validity (*i.e.* fully represent each construct by reflecting all its subconstructs).

#### External expert review of candidate measures

We invited a convenience sample of 15 measurement experts with knowledge of indicator development and the ‘re-visioning’ EmONC project objectives to review the candidate measures. Respondents were global and country-level measurement developers or implementers from global guidance agencies, academia, international non-governmental organisations, or EmONC facilities based in LMICs. Measures of individual and team well-being constructs were evaluated separately. For each construct (**Table 2**, **Table 3**), we asked the reviewers to quantitatively score the quality of the candidate measures against the prespecified evaluation criteria (**Box 2**), and then to designate the stronger of the two measures presented for each subconstruct, to be combined into their ‘preferred composite scale’ for each construct.

Finally, to help with the ultimate selection, we asked the external reviewers for an overall qualitative assessment of the candidate composite scales. Specifically, we asked them to assess each construct’s appropriateness as a proxy or tracer for overall well-being and the relative strength of their ‘preferred scale’ to measure each construct.

#### Final selection of measures

For each candidate measure, RRJ, IG, and EM calculated a total score based on its rating by the prespecified measure quality criteria and how often respondents selected it as their preferred measure for the given subconstruct. We then used these scores to prioritise the highest-scoring measures for inclusion. Qualitative feedback and the final ranking of the ‘preferred scales’ for individual and team well-being constructs guided the final selection of composite measures for these constructs: burnout, moral distress, and job strain (individual well-being), and psychological safety and role clarity (team well-being). We compiled short scales for these constructs from the subconstruct measures with the highest scores.

Then, as we aimed to identify and prioritise no more than three tracer indicators for health worker well-being, the research team deliberated to remove two of the five finalist measures. We chose job strain, defined as high demand and low control at work, for elimination because a central component of the construct (*i.e.* high demand) was operationalised as insufficient human resources; we reasoned that staffing levels are already slated for measurement in the revised EmONC framework. Therefore, although job strain was deemed highly important and relevant, a key determinant of this construct will already be routinely monitored.

We next eliminated role clarity based on suboptimal fit with our *a priori* conceptualisation of well-being, which focusses on its affective and subjective dimensions. We reasoned that this construct, as defined in the literature [93–104], reflects health system functionality and team performance more so than affective well-being relative to the other candidate measures.

Thus, after our rigorous, structured measure development process focussed on construct validity and measure quality, we were left with three candidate measures for the constructs of burnout, moral distress, and psychological safety.

The final step was to transform them for use as a set of tracer facility-level indicators. To achieve this, we specified harmonised metadata and developed a summative score and target values for each short scale. We then articulated aggregated facility-level indicators derived from these data, which we propose can serve as tracer indicators for overall EmONC health worker well-being.

#### Final measure development

To meet the overall study objective of a brief set of tracer indicators suitable for routine monitoring at the facility and district levels, we made minor revisions to harmonise the metadata across the set (Table S1 in the **Online Supplementary Document**). First, we modified the time frame for all time-bound items to ‘within the last month’ to match other indicators in the revised EmONC framework. Second, because responses from Likert-type scales using differing response formats are not comparable and their interpretation is *de facto* relative, we harmonised the response options to be consistent across all the items. Based on evidence examining the relationship between scale formats and their effect on various types of bias, we chose to harmonise the response options for all items to a five-point intensity scale, labelled as 1 – not at all, 2 – very little, 3 – moderately, 4 – a lot, and 5 – extremely. This five-point intensity scale format has been linked to reduced acquiescence bias, fewer extreme responses, less incorrect answering of reversed questions, and better criterion validity [105,106]. In addition, it mirrors the original scale used for the three selected burnout measures.

Finally, we set target scores and articulated aggregate facility-level indicators for the measures. Indicators in the ‘re-visioning’ EmONC framework serve to raise a ‘red flag’ when critical factors of emergency readiness are not met. In setting targets for monitoring health worker well-being, we propose that average values for burnout and moral distress should remain in the bottom two quartiles (no more than ‘moderately’), and average psychological safety should remain in the top two quartiles (no less than ‘moderately’). Thus, applying score values of 0–4 to the five-point Likert-type scales, average scores for the negatively-framed constructs should remain ≤2, and a score of >2 raises a ‘red flag’. Although psychological safety is a positively framed construct, for consistency across the three measures, we propose that the indicator for psychological safety should monitor a ‘lack of psychological safety’; thus, average scores of <2 will raise a ‘red flag’.

To transform the measures into facility-level indicators, the same threshold values apply, and the indicator is expressed as the percentage of health workers employed in the facility whose average score is outside the cutoff for each of the three constructs.

## RESULTS

The final proposed measures are expressed as facility indicators for each construct (**Table 4**). The short scales for burnout and psychological safety are composed of one item to reflect each of the subconstructs included in our operational definitions of those constructs (**Table 2**, **Table 3**), thus demonstrating content validity. The proposed measure for moral distress exists as a validated single item in which the operational definition is included within the question.

**Table 4:** Final list of proposed indicators

	Variables	Response options	Numerator	Denominator	Periodicity	Disaggregation factors
**Health worker burnout**	Proportion of health workers with an average score >2 on the following scale: 1. During the past month I have felt: Emotionally exhausted at work. 2. During the past month I have felt: Physically exhausted at work. 3. During the past month my job has contributed to me feeling: Less sensitive to others’ feelings/emotions	Not at all (score = 0); very little (score = 1); moderately (score = 2); a lot (score = 3); extremely (score = 4)	Number of health workers in a facility with an average score greater than two (>2) on the burnout scale	Total number of health workers in a facility	Annual	Facility type (facility acuity level, EmONC designation, private/public sector); geographic area; health worker cadre
**Health worker moral distress**	Proportion of health workers with a score >2 on the following survey question: 1. Moral distress occurs when you believe you know the ethically correct thing to do, but something or someone restricts your ability to pursue the right course of action. Please select the response on the Moral Distress Scale that best describes how much moral distress you have been experiencing related to work in the past month, including today	Not at all (score = 0); very little (score = 1); moderately (score = 2); a lot (score = 3); extremely (score = 4)	Number of health workers in a facility with a score greater than two (>2) on the Moral Distress Scale	Total number of health workers in a facility	Annual	Facility type (facility acuity level, EmONC designation, private/public sector); geographic area; health worker cadre
**Health worker team psychological safety**	Proportion of health workers with an average score <2 on the following scale: 1. On this team, all team members feel safe to speak up if they see something that may negatively affect patient care. 2. On this team, if I made a mistake, I would feel safe speaking up to my team leader. 3. On this team, it is easy for all team members to ask questions when there is something that they do not understand	Not at all (score = 0); very little (score = 1); moderately (score = 2); a lot (score = 3); extremely (score = 4)	Number of health workers in a facility with an average score of less than two (<2) on the Psychological Safety Scale	Total number of health workers in a facility	Annual	Facility type (facility acuity level, EmONC designation, private/public sector); geographic area; health worker cadre

EmONC – emergency obstetric and newborn care

## DISCUSSION

We followed a rigorous, systematic five-phase process [107] encompassing two overall stages – construct and measure definition – to identify and prioritise three facility-level indicators that can be tested and validated for use as tracer measures of EmONC health worker well-being as part of the revised EmONC framework.

The SDGs vastly broadened the scope of the global development agenda from the previous framework, bringing focussed attention to social and systemic determinants of health and greatly increasing the number of health-related goals and targets [108–110]. At the advent of the SDGs, advocates issued calls to improve MNH measurement, setting a ‘measurement agenda’, and proposing principles of focus, relevance, innovation, equity, and leadership to guide it [52,111,112]. A plethora of measures ensued, with a systematic review published in 2019 identifying 1445 discrete MNH indicators [55]. Nevertheless, measures of individual and team MNH health worker well-being are still lacking.

Although we were unable to conduct a posteriori empirical testing, we paid close attention *a priori* to construct validity in this measure development activity, centring the foundational importance of ‘meaning’ and ‘meaningfulness’ in ensuring valid measures [52]. Recent indicator research in LMICs has demonstrated that many common health system-level indicators do not deliver conceptual clarity and showed that when measure definitions fail to capture the concept they intend to reflect, accurately or fully, loss of essential meaning constitutes a threat to validity [53]. To address this gap, therefore, during the first stage aimed at construct definition, we conducted systematic search for constructs related to workforce well-being for measurement and critically interpreted and synthesised the subconstructs to fully reflect the multidimensional definitions of those constructs. We considered both positively and negatively framed constructs related to health worker well-being. We further collected evidence on the meaningfulness of these constructs to end users through qualitative inputs provided by MNH stakeholders.

In the second stage, focussed on measure definition, we generated a broad pool of candidate items that have been previously validated and used to measure all dimensions of those constructs included in our operational definitions, combed from the literature. At this stage, we centred the ‘measurement’ and ‘measurability’, two additional aspects of indicator validity highlighted by Benova and colleagues in their definitional framework, which describe a measure’s technical soundness and its practicality, and the need to balance them [52]. At this stage, we systematically mapped, evaluated, and prioritised the candidate items. To balance quality and feasibility of the measures, we drew on the literature and inputs from content experts (*i.e.* MNH health workers and other stakeholders) as well as measurement experts, to keep the measurement goal of a brief, relevant, actionable set of tracer measures for monitoring health worker well-being at facility level at the forefront of our selection process. Items were chosen based on clarity, sensitivity to change, interpretability, usefulness, generalisability, and reliability, as well as parsimony, given the end goal of aggregation to the facility or district level.

To address the issue of measurability, our next step was to construct a brief measure set consisting of two small scales and one single-item measure from these prioritised items, to be calculated using individual health worker survey data. We considered the implications of various choices for harmonising the item format, response options, labels, and time frame [105–107]. Lastly, focusing on issues of measurement, we proposed metadata to transform these measures into a small number of facility-level indicators that can serve as proxy measures for routinely monitoring overall facility-based health worker well-being. In so doing, this effort addresses a significant gap since our search identified no available facility-level indicators to directly reflect the lived experience of health workers. Psychometric testing and validation are required, but were not feasible within the budget and timeline for this activity.

While recognising that three prioritised constructs cannot reflect the complex, multidimensional domain of health worker well-being in its entirety, those selected were assessed as being meaningful, relevant, and actionable by health care decision makers in LMIC contexts via stakeholder consultation. Further, the measures proposed for each construct fully represent the construct’s multiple dimensions synthesised from the literature. Thus, theoretically grounded in construct and content validity and reflecting all four components of the most common definitions of indicator validity, we propose that there is a strong conceptual basis for using these measures as tracers for overall health worker well-being in the facility setting. Furthermore, criterion validity is reflected in the fact that these measures collect self-reported data, the ‘gold standard’ for subject experience [52,55].

As next steps, the measures should be piloted and data collected using a minimum sample size of 70–105 subjects (*i.e.* 10–15 times the number of items) [113,114]. Confirmatory factor analysis is necessary to ensure that the items selected load onto each of the hypothesised constructs identified and that they are sufficiently distinct from each other. In addition, psychometric testing for internal consistency reliability using Cronbach’s alpha should be conducted. Finally, additional research should test for convergent (and possibly divergent) validity to explore whether scale scores converge with other measures one would expect them in theory to trend with (*e.g.* burnout and sick days), and whether they diverge from measures with which one would expect no association (*e.g.* moral distress and age).

The three final constructs cover a range of subjective experiences relevant to facility EmONC in LMICs. Burnout is a complex, multidimensional, physiological, and psychological syndrome arising in the context of chronic, unabated workplace stressors [67], and commonly characterised by emotional exhaustion, physical fatigue, and depersonalisation [68-71]. Moral distress is defined as the result of perceived pressure to act unethically with little power to change the situation. It may occur when professional standards of care are not met due to institutional constraints [76,77]. Psychological safety is defined as the perception that it is safe to take interpersonal risks within a team, including openly disagreeing with other members of the team or ones’ superiors, particularly when errors or risks are identified [84–87], thus allowing for errors to be identified by any member of the team, and learned from [88]. Following psychometric testing and validation, these candidate indicators can provide valuable data on important constructs of health worker well-being at the facility level. They suggest actionable solutions and raise a proverbial ‘red flag’ when the values indicate well-being in these domains is low, in keeping with other critical dimensions of emergency service readiness and experience of care that the revised EmONC framework aims to monitor.

This study has significant implications for practice. Recent literature has found that health worker well-being, patient outcomes, and organisational change are highly correlated [51], indicating that health worker well-being is important to measure. Burnout in the health care setting, for example, is associated with sleep deprivation [115], medical errors [116–118], poor quality of care [119,120], and low patient satisfaction [121]. High rates of burnout are significantly associated with health worker attrition in numerous studies [122–125], worsening existing critical shortages. Additional literature suggests that frequent moral distress can lead to burnout or leaving the profession altogether [126–128]. Furthermore, a lack of psychological safety can inhibit health workers and result in avoidance behaviours such as silence and withholding ideas, suggestions, or concerns [129,130]. Although much of the currently published literature comes from high-income countries, lack of well-being among health workers in LMICs is likely both more prevalent, given known system deficiencies, and associated with similar outcomes. Nonetheless, a review of the existing peer-reviewed and grey literature on health worker well-being demonstrated that there is no standard approach to defining or measuring it at the facility or district level. The proposed measures fulfil the criterion of ‘usefulness’, meaning they point to a response from organisational leadership. Thus, tracking health workers' well-being by monitoring these constructs and addressing identified problems could improve the quality of care as well as staff retention and motivation. Data collection and reporting would need to be sensitive to issues of confidentiality and potential retaliation.

A recent Cochrane review found little knowledge about routine health information system coverage and use of data for health system improvement beyond the clinical sphere (*e.g.* to guide governance and human resource management) [131]. There is interest in better harnessing the power of routine health information systems for health system strengthening, including to ensure that the system’s ‘software’ (*i.e.* human factors including relationships and power, values and norms, ideas and interests) [132] is running smoothly [133]. Once validated in LMICs through piloting and psychometric testing, we hope that tracking this set of tracer measures of well-being through routine data collection and monitoring them at the facility and district levels will enable health system administrators and decision makers to identify ‘red flags’ and respond with actionable solutions on time. We hypothesise that doing so will improve both health workers’ well-being and, as a result, impact patient outcomes via improved quality and experiences of care.

### Strengths and limitations

There are several strengths to our study. To our knowledge, we are the first to attempt to identify a brief set of facility-level indicators for routinely monitoring health worker well-being in LMICs. Another strength is the systematic approach used in identifying important constructs and developing measures of both individual and team well-being, as well as our careful documentation of the process. Throughout the process of selecting constructs and defining measures for identified constructs, we focussed on the relevance and importance to key stakeholders, emphasising construct, concept, and criterion validity to ensure meaning and meaningfulness.

However, several limitations should be noted. We were unable to identify existing facility-level indicators comprised of data that are easily collected via routine information systems. In addition, most available measurement tools for health worker well-being exist as long survey questionnaires or direct observation tools that are inappropriate for routine measurement and thus infeasible for our current purpose. Long surveys require personnel with special skills to administer them, are time-consuming, and require more resource utilisation to compile and analyse results. Consequently, they do not lend themselves to routine facility tracking. Therefore, to develop a set of no more than three tracer indicators to serve as proxies for overall well-being, we were obliged to re-group single items from existing validated surveys into small scales, and to modify the validated single-item measure for the third construct from a visual analogue format. We acknowledge that self-administered scales are subject to some limitations, including low response rates, error, and misinterpretation, as well as courtesy bias if health workers adapt their responses for fear of retribution from the institution. However, these limitations are not specific to our study, but rather to the goal of routinely monitoring the subjective experience of health workers with criterion validity.

Another limitation is that most of the literature on health worker well-being and tools for its measurement comes from high-income countries. These limitations imply that our proposed measures will require pilot testing to collect data with which to conduct psychometric and cognitive testing for further validation. Lastly, although we used a systematic approach throughout the identification and analysis process, our methods and the validity of our results were limited by time and resource constraints.

## CONCLUSIONS

Health worker well-being is foundational to a person-centred approach to health care delivery, yet its measurement is not part of the routine monitoring of facility functionality. We developed three indicators based on a broad scoping review of the literature and expert consultations with content and measurement experts to indicate high levels of burnout and moral distress and low levels of psychological safety among MNH health workers in EmONC facilities. These indicators are proposed as proxies for overall health worker well-being, acting as ‘red flags’ in keeping with other indicators in the EmONC framework, to alert health care system administrators and decision makers of the need to address root causes of threats to worker well-being. The proposed indicators require pilot testing and empirical validation, including cognitive and psychometric testing, before they can be fully integrated into the EmONC framework.

## Additional Material


Online Supplementary Document
