# Supplementary material for: The development of proxy indicators to measure workforce well-being in emergency obstetric and neonatal care settings
Source: J Glob Health. 2025 Aug 10;15:04223. doi: 10.7189/jogh.15.04223 (PMC12427604; doi:10.7189/jogh.15.04223)
Supplement: Online Supplementary Document [file jogh-15-04223-s001.pdf]

Supplement to: Jolivet RR, Munson E, Gouse I, Chodzaza E, Chowdhury ME, Dieng T, Lobis S, Moreira I, Ramsey K, Warthin C, Freedman L. Proxy Indicators to Measure Workforce Well-being in EmONC Settings. J Glob Health. 2025;15:04223.

Box S1. Search strategy for constructs of individual health worker well-being

((((health workforce[MeSH Terms]) OR (health personnel[MeSH Terms]) AND ((y_10[Filter]) AND (meta-analysis[Filter] OR review[Filter]))) AND (((((((Burnout, Professional / etiology[MeSH Terms]) OR (Burnout, Professional / psychology[MeSH Terms])) OR (Cooperative Behavior[MeSH Terms])) OR (Nurse's Role / psychology[MeSH Terms])) OR (Nursing Staff / psychology*[MeSH Terms])) OR (Personnel Turnover[MeSH Terms])) OR (Professional Autonomy[MeSH Terms]) AND ((y_10[Filter]) AND (meta-analysis[Filter] OR review[Filter])))) AND (((((Health Facility Environment[MeSH Terms]) OR (Salaries and Fringe Benefits[MeSH Terms])) OR (Workplace / organisation & administration[MeSH Terms])) OR (Workplace / psychology[MeSH Terms])) OR (Community Health Workers / psychology*[MeSH Terms]) AND ((y_10[Filter]) AND (meta-analysis[Filter] OR review[Filter])))) AND ((wellbeing) OR (well-being))

Box S2. Search strategy for constructs of team health worker well-being

((maternal OR obstetric OR neonatal) AND teamwork) AND (professional AND interprofessional collaboration OR professional collaboration OR interprofessional collaboration OR worker collaboration OR team collaboration OR professional cooperation OR worker cooperation OR team cooperation OR role clarity OR team clarity OR clear job description* OR clear roles OR mutual role understanding OR team role understanding OR team agreement OR professional boundaries OR culture of patient safety OR team safety OR effective communication OR team communication OR conflict management OR team conflict OR mutual trust OR professional trust OR worker trust OR team trust) AND (well-being OR wellbeing)

Table S1. Original and proposed measures for burnout, moral distress, and psychological safety

Reference	Measurement Tool	Original Item	Proposed Item	Original Response Options	Proposed Response Options
**Burnout**					
Trockel et al., 2018 [1]	Stanford Professional Fulfillment Index	During the past two weeks I have felt: Emotionally exhausted at work.	*During the past month I have felt: Emotionally exhausted at work.*	Not at all, Very little, Moderately, A lot, Extremely	Not at all, Very little, Moderately, A lot, Extremely
		During the past two weeks I have felt: Physically exhausted at work.	*During the past month I have felt: Physically exhausted at work.*		
		During the past two weeks my job has contributed to me feeling: Less sensitive to others' feelings/emotions.	*During the past month my job has contributed to me feeling: Less sensitive to others' feelings/emotions.*		
**Moral Distress**					
Wocial et al., 2013 [2]	Moral Distress Thermometer	Moral distress occurs when you believe you know the ethically correct thing to do, but something or someone restricts your ability to pursue the right course of action. Please circle the number (0-10) on the Moral Distress Thermometer that best describes how much moral distress you have been experiencing related to work in the past week including today.	*Moral distress occurs when you believe you know the ethically correct thing to do, but something or someone restricts your ability to pursue the right course of action. Please select the response on the Moral Distress Scale that best describes how much moral distress you have been experiencing related to work in the past month, including today.*	Intensity scale from 1-10 (10/ Worst possible, 8/ intense, 6/ distressing, 4/ uncomfortable, 2/ mild, 0/none)	Not at all, Very little, Moderately, A lot, Extremely

**Psychological Safety**					
Agency for Healthcare Research and Quality (AHRQ) [3]	SOPS Hospital Survey 2.0	In this unit, staff speak up if they see something that may negatively affect patient care.	*On this team, all team members feel safe to speak up if they see something that may negatively affect patient care.*	Never, Rarely, Sometimes, Most of the time, Always, Does Not Apply or Don't Know	Not at all, Very little, Moderately, A lot, Extremely
O'Donovan et al., 2020 [4]	Psychological safety in healthcare teams	If I made a mistake on this team, I would feel safe speaking up to my team leader.	*On this team, if I made a mistake, I would feel safe speaking up to my team leader.*	7-point Likert scale from strongly disagree to strongly agree	
Hutchinson et al., 2006 [5]	Safety attitudes and safety climate questionnaire	It is easy for personnel here to ask questions when there is something that they do not understand.	*On this team, it is easy for all team members to ask questions when there is something that they do not understand.*	5-point Likert scale from strongly disagree to strongly agree	

Table S2. Proposed indicators for health worker well-being

<table>
<tr><td colspan="5">Health worker burnout

Proportion of health workers with an average score >2 on the following scale:

1. During the past month I have felt: Emotionally exhausted at work.
2. During the past month I have felt: Physically exhausted at work.
3. During the past month my job has contributed to me feeling: Less sensitive to others’ feelings/emotions.</td></tr>
<tr><td><u>Response Options</u>

● Not at all (score = 0)
● Very little (score = 1)
● Moderately (score = 2)
● A lot (score = 3)
● Extremely (score = 4)</td><td><u>Numerator</u>

Number of health workers in a facility with an average score greater than two (>2) on the burnout scale</td><td><u>Denominator</u>

Total number of health workers in a facility</td><td><u>Periodicity</u>

Annual</td><td><u>Disaggregation Factors</u>

● Facility type (facility acuity level, EmONC designation, private/public sector)
● Geographic area
● Health worker cadre</td></tr>
<tr><td colspan="5"></td></tr>
<tr><td colspan="5">Health worker moral distress

Proportion of health workers with a score >2 on the following survey question:

1. Moral distress occurs when you believe you know the ethically correct thing to do, but something or someone restricts your ability to pursue the right course of action. Please select the response on the Moral Distress Scale that best describes how much moral distress you have been experiencing related to work in the past month, including today.</td></tr>
<tr><td><u>Response Options</u>

● Not at all (score = 0)
● Very little (score = 1)
● Moderately (score = 2)
● A lot (score = 3)
● Extremely (score = 4)</td><td><u>Numerator</u>

Number of health workers in a facility with score greater than two (>2) on the Moral Distress Scale</td><td><u>Denominator</u>

Total number of health workers in a facility</td><td><u>Periodicity</u>

Annual</td><td><u>Disaggregation Factors</u>

● Facility type (facility acuity level, EmONC designation, private/public sector)
● Geographic area
● Health worker cadre</td></tr>
<tr><td colspan="5"></td></tr>
</table>

Health worker team psychological safety

Proportion of health workers with an average score <2 on the following scale:

1. On this team, all team members feel safe to speak up if they see something that may negatively affect patient care.
2. On this team, if I made a mistake, I would feel safe speaking up to my team leader.
3. On this team, it is easy for all team members to ask questions when there is something that they do not understand.

Response Options	Numerator	Denominator	Periodicity	Disaggregation Factors
● Not at all (score = 0)<br>● Very little (score = 1)<br>● Moderately (score = 2)<br>● A lot (score = 3)<br>● Extremely (score = 4)	Number of health workers in a facility with average score less than two (<2) on the Psychological Safety Scale	Total number of health workers in a facility	Annual	● Facility type (facility acuity level, EmONC designation, private/public sector)<br>● Geographic area<br>● Health worker cadre

References

1 Trockel M, Bohman B, Lesure E, Hamidi MS, Welle D, Roberts L, et al. A Brief Instrument to Assess Both Burnout and Professional Fulfillment in Physicians: Reliability and Validity, Including Correlation with Self-Reported Medical Errors, in a Sample of Resident and Practicing Physicians. Acad Psychiatry. 2018;42:11–24.

2 Wocial LD, Weaver MT. Development and psychometric testing of a new tool for detecting moral distress: the Moral Distress Thermometer. J Adv Nurs. 2013;69:167–74.

3 Agency for Healthcare Research and Quality. Hospital Survey on Patient Safety Culture. 2013. Available: https://www.ahrq.gov/sops/surveys/hospital/index.html. Accessed: 18 September 2023.

4 O'Donovan R, McAuliffe E. Exploring psychological safety in healthcare teams to inform the development of interventions: combining observational, survey and interview data. BMC Health Serv Res. 2020;20:810.

5 Hutchinson A, Cooper KL, Dean JE, McIntosh A, Patterson M, Stride CB, et al. Use of a safety climate questionnaire in UK health care: factor structure, reliability and usability. Qual Saf Health Care. 2006;15:347–53.
